# The burden of healthcare-associated infections in New Zealand public hospitals 2021

**DOI:** 10.1017/ice.2024.95

**Published:** 2024-10

**Authors:** Arthur J. Morris, Mike Hensen, Nicholas Graves, Yiying Cai, Martin Wolkewitz, Sally A. Roberts, Nikki Grae

**Affiliations:** 1 Infection Prevention & Control Programme, Health Quality & Safety Commission, Auckland, New Zealand; 2 New Zealand Institute of Economic Research, Wellington, New Zealand; 3 Health Services and Systems Research, Duke-National University of Singapore Medical School, Singapore, Singapore; 4 Institute of Medical Biometry and Statistics, Division Methods in Clinical Epidemiology, Faculty of Medicine and Medical Center, University of Freiburg, Freiburg, Germany

## Abstract

**Background:**

There are no contemporary data on the burden of healthcare-associated infections (HAIs) in New Zealand.

**Objectives:**

To estimate the economic burden of HAIs in adults in New Zealand public hospitals by number and monetary value of bed days lost; number of deaths, number of life years lost, and the monetary value (in NZ dollars); Accident Compensation Commission (ACC) HAI treatment injury payments; and disability-adjusted life years (DALYs).

**Methods:**

The annual incidence rate was calculated from the observed prevalence of HAIs in New Zealand, and length of patient stays. Total HAIs for 2021 were estimated by multiplying adult admissions by incidence rates. The excess length of stay and mortality risk attributed to those with HAI was calculated using a multistate model. Payments for treatment injuries were obtained from the ACC. DALYs for HAIs were estimated from the literature.

**Results:**

The incidence rate of HAI was 4.74%, predicting 24,191 HAIs for 2021, resulting in 76,861 lost bed days, 699 deaths, with 9,371 years of life lost (YoLL). The annual economic burden was estimated to be $955m comprised of $121m for lost bed days, $792m for cost of YoLL, and $43m ACC claims. There were 24,165 DALY which is greater than many other measured injuries in New Zealand, eg motor vehicle traffic crashes with 20,328 DALY.

**Conclusions:**

HAIs are a significant burden for patients, their families, and the public health system. Preventive guidelines for many HAIs exist and a strategic plan is needed to reduce HAIs in New Zealand.

## Introduction

Healthcare-associated infections (HAIs) are a significant public health problem associated with increased morbidity, mortality, length of stay (LoS), healthcare, and socioeconomic cost.^1–8^ There is limited information from New Zealand on the prevalence, incidence rates, and economic burden of HAIs. Previous point prevalence surveys (PPS) in Auckland District Health Board (DHB) hospitals in the late 1990s reported a HAI incidence of 6.3%, ie 6.3 patients per 100 admissions, and a HAI prevalence of 9.5%.^9,10^ The estimated 1999 cost of HAI for Auckland DHB was almost $19 million, or $34m in 2021 prices, and for the country $137 million, or $247m in 2021 prices.^5^ A recent 2021 study found a prevalence of HAIs in adult patients in public hospitals in New Zealand to be 6.6%.^11^

A major economic impact of HAI is that scarce and valuable bed days are used to manage the consequences of HAI because a case of HAI prolongs stay or requires readmission, in either a ward or ICU bed. Valuing these bed days can be done with retrospective accounting data that show the average historical spend per bed day or, decision-makers can be asked prospectively about their willingness to pay to release bed days from infection prevention.^12^ There are advantages and disadvantages with either approach, but on balance, the willingness to pay approach will provide economic values suitable for future decision-making.^13^

Another major consequence is that lives are lost as the HAI increases risk of mortality in hospital, denying patients years of productive life in the future. The indirect economic effects of HAI can also be substantial with patients needing time to get back to employment and their usual activities.^14^ They may additionally incur costs after discharge from incidental use of primary care and community-based health services.^15^ New Zealand has a unique no-fault scheme for injury compensation administered by the Accident Compensation Commission (ACC).^16^ For selected groups and outcomes, including HAI, weekly compensation for lost earnings and lump sum payments, as well as funds for treatment and rehabilitation costs are provided to cover the individuals affected. Recent legislative changes have brought forward the date of access to the minimum rate of injury compensation from six weeks to two, increasing the public cost of HAI.

The aim of this study is to estimate the economic burden of HAI arising in New Zealand public hospitals. The economic burden we report includes the number and monetary value of bed days lost, the number of deaths, the number of life years and monetary value lost, and the payments made by the ACC for victims of HAI. These are summed to show an aggregate national economic burden of HAI. We also estimated, by a separate process, the burden of HAIs measured by disability-adjusted life years (DALYs) to allow comparisons with other conditions and injuries reported in New Zealand.

## Methods

**Figure 1.**
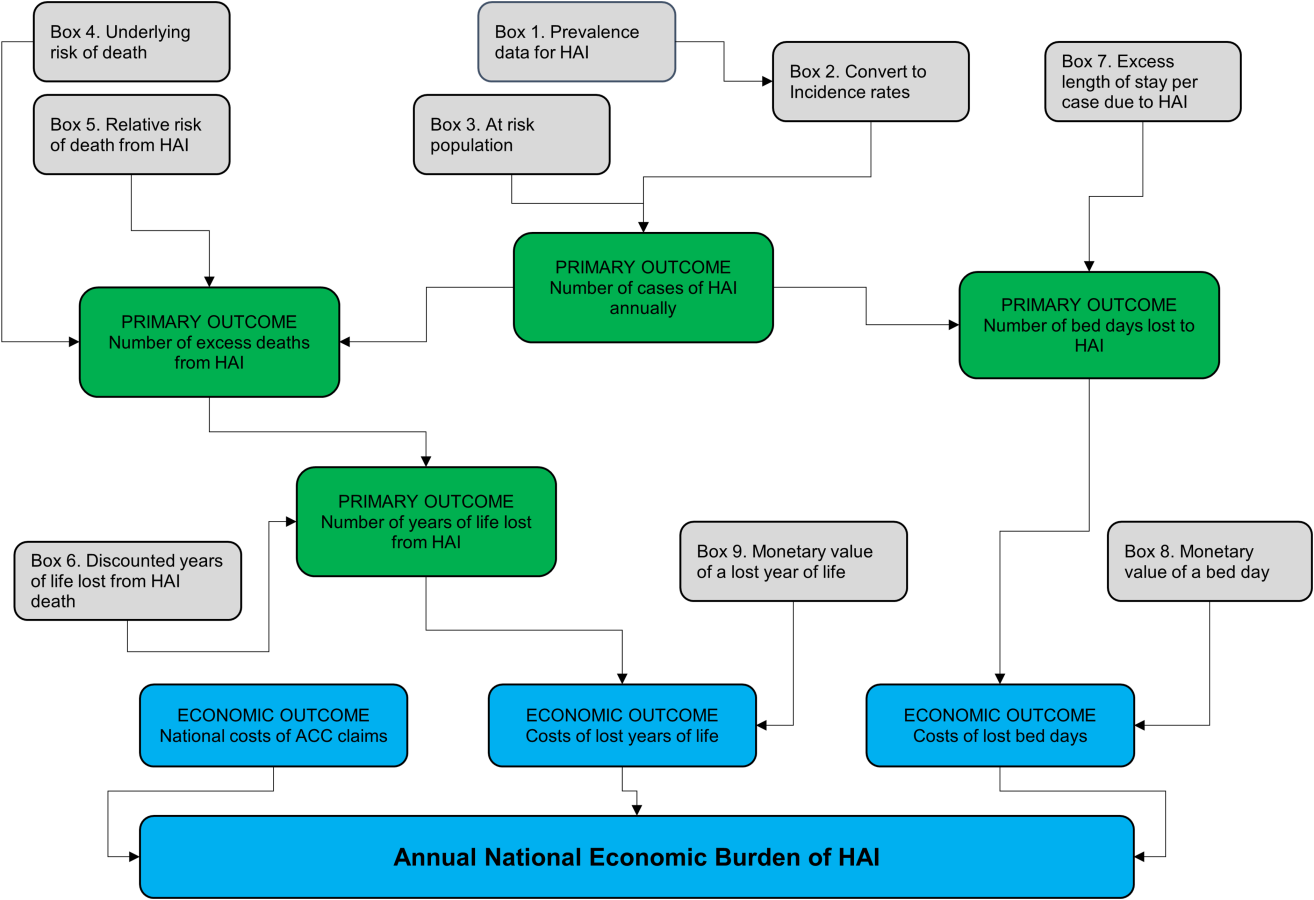
Overview of the quantitative model used to estimate economic outcomes.

The entire population of adult admissions for 2021 to public hospitals in New Zealand was included (n = 510,289). A quantitative model was developed to estimate the economic burden of HAI using a range of data sources. An overview of the model is provided in Figure 1.

Four primary outcomes shown as green boxes in Figure 1 are estimated: number of cases of HAI annually; number of excess deaths from HAI; number of years of life lost (YoLL) from HAI; and number of bed days lost to HAI. These primary outcomes arise from combining the data inputs shown as gray boxes. The final economic outcomes are shown as blue boxes. The “national costs of ACC claims” are based on reported data.^16^ The outcomes of “Costs of YoLL” and “Costs of lost bed days” arise from combining the primary outcomes (green boxes) and data inputs (gray boxes). The aggregate economic burden of HAI is the sum of the three economic outcomes (blue boxes). All monetary values are in New Zealand dollars (NZD).

### Estimating primary outcome - number of cases of HAI annually

This was informed by using prevalence data to estimate the incidence of HAIs (Figure 1, Boxes 1 and 2). Between 22 February and 23 June 2021, a point prevalence survey was conducted across all DHB acute care public hospitals using the European Centre for Disease Prevention and Control (ECDC) methodology and HAI definitions.^11^ There were 5,468 patient records surveyed and this represented 3% of the discharges for the survey period. The prevalence data were matched with the National Minimum Data Set to obtain the discharge dates used to generate the estimation of incidence rates; with 5,353 patients matched successfully. The prevalence of all HAI was 6.31%. The Rhame and Sudderth formula^17^ was then used to estimate cumulative incidence rates of HAI. It is,

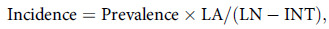




where LA is the length of stay of all hospitalized patients, irrespective of the presence of an HAI, LN is the length of stay of patients with an HAI, and INT is the length of stay before the onset of the HAI. The incidence rates by type of infection, and the information used to establish the associated model parameters, are shown in the supplementary material (Table S1 – part A).

The incidence rate for HAI was calculated to be 4.74% patients per 100 admissions for 2021. The at-risk population (Figure 1, Box 3) was 510,289 adults admitted to public hospitals in 2021.

### Estimating primary outcome - number of excess deaths from HAI

This outcome depended on the number of cases of HAI annually, the underlying risk of death (Figure 1, Box 4), and the relative risk of death from HAI (Figure 1, Box 5). Underlying risk of death was moderated by the relative risk of death from having an HAI. We made two-by-two tables for each HAI type including those with HAI, those without HAI, those discharged alive, and those discharged dead. Relative risk (RR) of death, and the variance, was estimated from the data in the tables. The log RR was taken and used to update a normal distribution. Monte Carlo simulations (n = 1000) were performed and for each resample the exponent was used to update the results for the model presented.

The excess risk of death was applied to the number of HAI cases annually. The data used to establish these model parameters were taken from the PPS^11^ and are shown in the supplementary material (Table S1 - Part B). To address the effects of length-biased sampling, we utilized weighting by replicating observations inversely proportional to their length of stay.^18^ Standard errors were calculated using generalized estimating equations.

### Estimating primary outcome - number of years of life lost from HAI

This outcome depended on the number of excess deaths from HAI and the discounted years of life lost from HAI death (Figure 1, Box 6). To estimate the 13.41 discounted years of life lost from an HAI death we used the mean age of the sample of 65 years, the NZ life expectancy of 81.75 years, and applied a discount rate of 3%.^19^

### Estimating primary outcome - number of bed days lost to HAI

This outcome depended on the number of cases of HAI annually and the excess length of stay per case due to HAI (Box 7). Traditional methods for estimating excess hospital stay suffer from time and length biases as they fail to treat HAI as a time-varying exposure. The result is an inevitable overestimation of length of stay.^20^ A solution is to use multistate models that account for time as a continuous occurrence. Multistate models also allow for competing risks of death and discharge.^21^ The state-based model we use is shown in Supplementary Figure S1 and is updated with information on dates of admission, onset of infection, and discharge to dead or alive observed in the PPS.^11^ Again, to account for length-biased sampling, we weighted observations inversely proportional to their length of stay and computed standard errors by clustered bootstrapping.^18^

The results from the state-based model and resulting parameters used for the economic model are shown in the supplementary material (Table S1 - Part C).

### Estimating economic outcome - costs of lost bed days

This depended on the primary outcome of number of bed days lost to HAI and the monetary value of a bed day (Figure 1, Box 8). Data were retrieved from Health New Zealand, Te Whatu Ora, Te Toka Tumai, Auckland district, previously known as Auckland DHB. Based on a nine-month period in 2022 the average cost for a general medical service bed was $1,569 and comprised: $370 for medical overhead; $932 for ward bed overheads; and $267 for laboratory and pharmacy costs.

### Estimating economic outcome - costs of YoLL

This depended on the primary outcome of number of years of life lost from HAI and the monetary value of a year of life (Figure 1, Box 9). The value of the per capita gross domestic product was used to approximate the value of a year of life lost. This is based on the recommendation to use 0.5 to 1.5 times GDP per capita as a basis to value a marginal quality-adjusted life year gained for decision-making against the paradigm of cost-effectiveness.^22^ The GDP per capita in NZ dollars for 2021 was $49,996 USD^23^ or $84,482 NZD.

### Estimating economic outcome - national costs of ACC claims

Costs compensated by ACC for HAI-related injuries for the financial year ending June 2020, were obtained from the ACC.^16^ There are three broad categories of entitlements a claim could receive: Compensation, weekly compensation for lost earnings, lump sums, and death benefits; Treatment, initial hospital treatment and on-going primary and secondary treatment; and Rehabilitation support physical rehabilitation and various forms of personal support. The total annual costs reported for each category are $10,504,922 for compensation, $6,210,922 for rehabilitation and $9,067,821 for treatment.^16^ We assume between 30% and 50% of HAI are unclaimed and adjust for this.

### HAI burden in DALY

The burden of a disease can be described using DALYs, a composite of quantifying the health losses in years by adding the number of years of health lost due to disability and the number of years of life lost due to premature death. We used a weighted average of DALY for five HAIs as calculated by Cassini et al based on European data.^3^ HAI associated DALYs were compared to DALY estimates for other diseases, conditions, and injuries in New Zealand.^24,25^

### Ethics

As an audit and related activity, the PPS study was determined by New Zealand’s Health and Disability Ethics Committee (HDEC) to be out of scope and did not require ethical committee review (Katz T. Personal communication to S.A. Roberts, August 14^th^, 2020, HDEC). This burden analysis was also out of scope for HDEC review (HDEC 6^th^ July 2022).

## Results

### Number of HAI, deaths and years of life and bed days lost

There were 510,289 adult admissions to public hospitals in 2020–21 and 4.74%, 4.74 patients per 100 admissions for 2021, of these, were incident cases of HAI. The breakdown by type of HAI for the number of cases, number of deaths, YoLL, and the number of bed days lost to HAI, from those who died and those who subsequently survived are shown in Table 1. For 2021 we estimated there were 24,191 HAIs, 699 deaths, and 76,861 lost bed days due to HAIs.

**Table 1.** Results for primary healthcare-associated infections (HAI) outcomes included in the quantitative model, for New Zealand adults 2021

HAI CASES	Mean (SD)	Median (IQR)	Min max
Bloodstream	2,857 (52.04)	2,856 (2822–2893)	2,685 – 3,032
Gastrointestinal	765 (28.40)	764 (746–785)	691 – 849
Lower respiratory	307 (16.73)	306 (295–318)	250 – 357
Surgical site	6,128 (80.39)	6,129 (6,075–6,184)	5,889 – 6,401
Urinary tract	4,590 (66.72)	4,592 (4,542–4,636)	4,403 – 4,793
Pneumonia	4,697 (67.26)	4,698 (4,654–4,742)	4,448 – 4,929
Other	4,847 (68.62)	4,847 (4,800–4,889)	4,601 – 5,055
Total	24,191 (154.82)	24,190 (24,085–24,290)	23,720 – 24,676
DEATHS	Mean (St Dev)	median (IQR)	min max
Bloodstream	100 (126.35)	58 (9–141)	0 – 1013
Gastrointestinal	81 (23.91)	78 (63–95)	29 – 189
Lower respiratory	35 (9.18)	34 (28–40)	13 – 72
Surgical site	0 (0.00)	0 (0–0)	0 – 0
Urinary tract	74 (138.26)	13 (0–89)	0 – 1,415
Pneumonia	225 (159.51)	193 (109–307)	0 – 0,864
Other	185 (145.41)	156 (78–255)	0 – 931
Total	699 (305.45)	642 (475–870)	125 – 2,319
YEARS of LIFE LOST (YoLL)	Mean (St Dev)	median (IQR)	min max
Bloodstream	1339 (1694.30)	780 (126–1890)	0 – 13,583
Gastrointestinal	1081 (320.68)	1044 (850–1279)	391 – 2,538
Lower respiratory	464 (123.04)	451 (377–535)	177 – 0,968
Surgical site	0 (0)	0 (0–0)	0 – 0,000
Urinary tract	0,989 (1,854)	0,168 (0–1,199)	0 – 18,973
Pneumonia	3,013 (2,139)	2,594 (1,468–4,111)	0 – 11,593
Other	2,484 (1,950)	2,090 (1041–3,419)	0 – 12,484
Total	9,371 (4,096)	8,605 (6,371–11,664)	1,676 – 31,099
BED DAYS	Mean (St Dev)	median (IQR)	min max
Bloodstream	11,775 (4606.72)	11,183 (8,385–14,298)	1379 – 32,892
Gastrointestinal	4,694 (2607.09)	4,245 (2,737–6,174)	247 – 15,234
Lower respiratory	2,257 (891.73)	2,131 (1,571–2,803)	455 – 5,510
Surgical site	9,745 (5438.64)	8,708 (5,726–12,427)	988 – 39,607
Urinary tract	11,993 (5755.73)	11,042 (7,814–15,374)	1179 – 38,877
Pneumonia	22,632 (6401.57)	22,051 (18,076–26,318)	8197 – 52,583
Other	13,765 (6239.36)	12,562 (9,286–17,382)	1551 – 36,948
Total	76,861 (12707.40)	76,114 (68,288–85,070)	40,473 – 133,161

The results for the three economic outcomes and the annual national economic burden (NZD) are shown in Table 2 and Supplementary Figure S2. The economic burden, $955 million, is dominated by the cost associated with YoLL, $792 million (83%). The cost for lost bed days was over $121 million.

**Table 2.** Economic outcomes on costs of healthcare-associated infections (HAI) for New Zealand (NZ) hospitals 2021, NZ dollars

	Mean (SD)	Median (IQR)	Min max
Costs of lost bed days	120,595,491 (19,937,908)	119,422,555 (107,143,688–133,475,124)	63,502,478 – 208,930,220
Costs of years of life lost	791,646,769 (346,044,930)	726,943,264 (538,261,462–985,407,410)	141,621,996 – 2,627,330,998
National costs of ACC claims	43,249,479 (4,483,691)	42,972,245 (39,065,677–46,878,813)	36,833,353 – 51,566,694
Total economic burden of HAI	955,491,738 (370,466,529)	889,338,064 (684,470,828–1,165,761,347)	241,957,827 – 2,887,827,912

### HAI burden in DALY

The estimate for DALYs, for the five HAIs covered in the analysis by Cassini et al,^3^ is 24,165, Table 3. These five HAIs comprise 79% of the estimated number of HAIs, 24,191, Table 3. The HAI DALY compared to other conditions and injuries in New Zealand is shown in Table 4. The DALY for HAIs is greater than most subdivisions condition groups reported, eg colorectal cancer, 24,012 DALY and all injury categories, eg motor vehicle traffic crashes, 20,328 DALY, and all workplace injuries 4,345 DALY.

**Table 3.** Total disability-adjusted life years (DALYs) for healthcare-associated infections (HAIs) in New Zealand 2021

HAI	N HAI^a^	Median DALY^b^	DALY total	% all DALY
Surgical site infection	6,128	0.37	2,267	9%
Urinary tract infection	4,590	0.53	2,433	10%
Pneumonia	4,697	1.23	5,777	24%
Bloodstream	2,857	4.51	12,885	53%
*Clostridioi des difficle* infection	765	1.05	803	3%
Totals	19,037		24,165	100%

a Table 1. Represents 79% of all HAIs (19,037/24,191).

b Median DALY for five HAIs was obtained from the analysis of a full data set from the European Centre for Disease Prevention and Control.^3^ Each HAI health outcome was based on decision trees developed from the global literature, ie death rate and rates of disability outcomes. For detail, see Supplementary Information in Cassini et al.^3^

**Table 4.** Disability-adjusted life years (DALYs) for conditions in New Zealand

	DALY	%	Reference
Total, all health conditions (2006)	955,000	100	
Selected Condition group subdivisions^a^			
Coronary artery disease	89,159	9.3	Ministry of Health, Health loss in New Zealand, 2006–2016. Reference 24.
Chronic obstructive respiratory disease	35,339	3.7	
Diabetes	28,808	3.0	
Colorectal cancer	24,012	2.5	
Traumatic brain injury	21,728	2.3	
Osteoarthritis	20,738	2.2	
Attributable burden (% DALY) for risk factors^b^			
High body mass index	75,445	7.9	
Alcohol	38,200	4.0	
High cholesterol	24,830	2.6	
All injuries total (2008)	83,808	100	
Injury category, examples			New Zealand estimates of the total social and economic cost of injuries. Reference 25.
Suicide and deliberate self-harm	21,548	26	
Motor vehicle traffic crashes	20,328	24	
Falls	10,424	12	
Workplace injuries	4,345	5	
Healthcare-associated infections (HAIs) (2021)^c^	24,165	100	Table 3, this report.

a This report summarizes the burden of disease and conditions in New Zealand for 2006–2016. Diseases or conditions are reported for 16 condition groups. Selected condition burdens are listed in Table 5, page 12 of the report. For the 30 subdivisions listed in Table 5 of reference 24, the HAI DALY was higher than 23 conditions (77%).

b Figure 21, page 36 of Ministry of Health report. Reference 24.

c 2021 DALY burden of HAIs in New Zealand is 24,165, Table 3. The five HAIs used to calculate cover 79% of all HAIs. If it is assumed that the remaining HAIs have a similar DALY the estimated total would be 30,548.

## Discussion

The annual economic burden of HAI in New Zealand public hospitals was estimated to be $955 million comprised of $121m for lost bed days, $792 million for cost of YoLL, and $43m ACC claims. The DALY burden of HAI was shown to be higher than many other conditions and causes of injury.

The excess LoS for HAIs has usually been calculated in a time-fixed way where the LoS without HAI is subtracted from the LoS with HAI.^26^ However, this approach overestimates the attributable LoS because the time before HAI onset is included as HAI time, this is recognized as time-dependant bias. To address the states an admission progresses through it is recommended that multistate modeling is used. The model accounts for discharge or death either with or without a HAI.^21,27^ The recent multistate analysis of excess LoS in Scotland was 7.8 days vs 27 days by simple LoS comparison between those with and without HAI, an overestimate of 3.5 times.^26^ We used a multistate model to avoid overestimation of excess LoS.

Our estimate for HAI-related deaths is similar to deaths observed in Europe using the same method to record HAI prevalence, 699 vs 919 respectively.^3^ Similarly, the population burden of DALY in Europe (501/10^5^ population) would estimate a total of 25,666 DALY (NZ population 2021, 5.123m), and we calculated 24,165 DALY.

While the cost of YoLL dominates in the economic burden of HAIs, the cost for lost bed days occupied by those with HAI was $121 million. Successful reduction in the incidence of HAI should free up bed days for the provision of other patient care. As fixed costs comprise most of hospital-associated HAI costs making beds available is the financial benefit of reducing HAIs rather than any reduction in variable costs.^2^

The New Zealand Ministry of Health publishes the burden of selected diseases and conditions.^24^ While the burden for amalgamated condition groups, eg cancers 167,149 DALY, is larger than the burden of HAIs, our estimated HAI DALY of 24,165 is greater than 23 of the 30 condition group subdivisions, eg colorectal cancer (24,012 DALY) and chronic kidney disease (7,360 DALY), Table 4.^24^ The burden of injuries, particularly deaths and injuries due to road traffic crashes, are recognized areas of concern in New Zealand. In 2022 there were 374 deaths due to road crashes and in 2008 the burden of motor vehicle crashes was 20,328 DALY.^25,28^ There were 699 HAI deaths estimated for 2021. The burden of HAI in New Zealand is greater than either traffic crash metric or all workplace injuries, both of which receive frequent public and political attention and legal enforcement for their reduction.

### This study has several strengths

The incidence is reliable being based on a prevalence determined by the well-validated ECDC method.^3^ Data were collected by a limited number of surveyors, who had high inter-observer agreement for recording HAIs.^11^ We mostly used observed data from our PPS for calculations^11^ as well as a multistate model to avoid overestimation of bed days lost.

### Our study has limitations

Our incidence was calculated from the prevalence of a single PPS.^11^ We used an estimate of the cost of a bed day based on one hospital’s medical bed day cost and whilst this would underestimate the daily cost of an intensive care bed, it may overestimate the daily cost in a less specialized hospital. We used HAI DALY from European data and, although based on decision trees based on the global literature, they may not be representative of New Zealand.^3^ ACC injury payments are recorded by claim description and not by a HAI surveillance definition. We used the Evaluation of Cost of Nosocomial Infection study data for gastrointestinal and lower respiratory tract HAIs as there were no local data available.^26,29^ We observed no deaths in those with surgical site infection and this would cause an underestimate of burden for this HAI. We assumed a level of underclaiming for ACC HAI-related injuries. We made no allowance for increased costs associated with multi-drug resistant organisms. Our findings describe the burden in New Zealand, it is not known how generalizable they are to other countries.

Infection prevention and control programs reduce HAIs with the strongest evidence for efficacy being for multimodal interventions, surveillance, monitoring, and feedback.^30,31^ Recently published strategies provide evidence-informed guidance for reducing HAIs.^32–35^ Our HAI burden analysis will inform a national strategy to reduce HAIs in New Zealand. Selection of where to focus will be based on relative burden, bed days lost, and DALY, as well as the relative effectiveness of intervention methods. However, given the increased frequency of *Staphylococcus aureus* intravenous catheter-associated bloodstream infections, and their associated mortality (9%), this is an obvious initial priority.^36,37^

## Conclusions

We have estimated the burden of HAIs by several metrics. The burden of HAI is greater than other well recognized causes of injury, ie road traffic crashes and workplace injuries. This underscores the need for a national infection prevention and control strategy to reduce HAIs in New Zealand. The best available body of evidence supports the use of care bundles alongside a multimodal implementation strategy as part of a nationally led infection prevention and control program.

## Supporting information

Morris et al. supplementary material 1 Morris et al. supplementary material

Morris et al. supplementary material 2 Morris et al. supplementary material

Morris et al. supplementary material 3 Morris et al. supplementary material

## Supplementary material

The supplementary material for this article can be found at https://doi.org/10.1017/ice.2024.95
