# Supplementary material for: The burden of healthcare-associated infections in New Zealand public hospitals 2021
Source: Infect Control Hosp Epidemiol. 2024 Oct 4;45(10):1176–82. doi: 10.1017/ice.2024.95 (PMC11611504; doi:10.1017/ice.2024.95)
Supplement: Morris et al. supplementary material 1 — Morris et al. supplementary material [file S0899823X24000953sup001.rtf]

Burden of HAI in New Zealand public hospitals. Supplementary material

Table S1. Model Parameters: Part A. Incidence rates by type of infection and the information used to establish the associated model parameters; Part B. Information used to estimate number of deaths from HAI; and Part C. Number of excess bed days used by type if HAI and patient outcome.

Supplementary Figure S1. Multistate models also allow for competing risks of death and discharge
Supplementary Figure S2. Annual national economic burden of HAI to New Zealand 


Table S1. Model Parameters: Part A. Incidence rates by type of infection and the information used to establish the associated model parameters; Part B. Information used to estimate number of deaths from HAI; and Part C. Number of excess bed days used by type if HAI and patient outcome.

A.	Incidence rates by type of infection and the information used to establish the associated model parameters	
HAI type	Incidence rate, %	Distribution used for uncertainty	
Bloodstream	0.56	Beta (714, 509,575)	
Gastrointestinal	0.15	Beta (612, 509,677)	
Lower respiratory	0.06	Beta (153, 510,136)	
Pneumonia	0.92	Beta (4440, 505,849)	
Surgical site	1.20	Beta (2551, 507,738)	
Urinary tract	0.90	Beta (4593, 505,696)	
Other	0.95	Beta (2705, 507,584)	
Total incidence 	4.74		
B.	Information used to estimate number of deaths from HAI	
Underlying relative risk (RR) of death	Estimate, %	Distribution used for uncertainty	
RR death Bloodstream	1.84	Beta (2,36)	
RR death Gastrointestinal a	4.94	Beta (24, 98)	
RR death Lower respiratory a	5.20	Beta (30, 115)	
RR death Pneumonia	2.54	Beta (5,64)	
RR death Surgical site	0		
RR death Urinary tract	1.13	Beta (2, 60)	
RR death Other	2.25	Beta (5,73)	
C.	Number of excess bed days used by type if HAI and patient outcome	
Excess days 	Days	Distribution used for uncertainty	
Bloodstream 	2.05	Gamma (6.62, 0.26)	
Gastrointestinal b	6	Gamma (3.11, 1.93)	
Lower respiratory b	7.3	Gamma (7.8, 1.07)	
Pneumonia 	2.42	Gamma (6.22, 0.39)	
Surgical site	1.53	Gamma (3.66, 0.42)	
Urinary tract	1.3	Gamma (2.09, 0.62)	
Other 	1.44	Gamma (2.56, 0.56)	
a Local data not available from PPS,11 estimates from ECONI study used25 
b Local data not available from PPS,11 estimates from ECONI study used26
