# Supplementary figures and images for: The burden of healthcare-associated infections in New Zealand public hospitals 2021
Source: Infect Control Hosp Epidemiol. 2024 Oct 4;45(10):1176–82. doi: 10.1017/ice.2024.95 (PMC11611504; doi:10.1017/ice.2024.95)

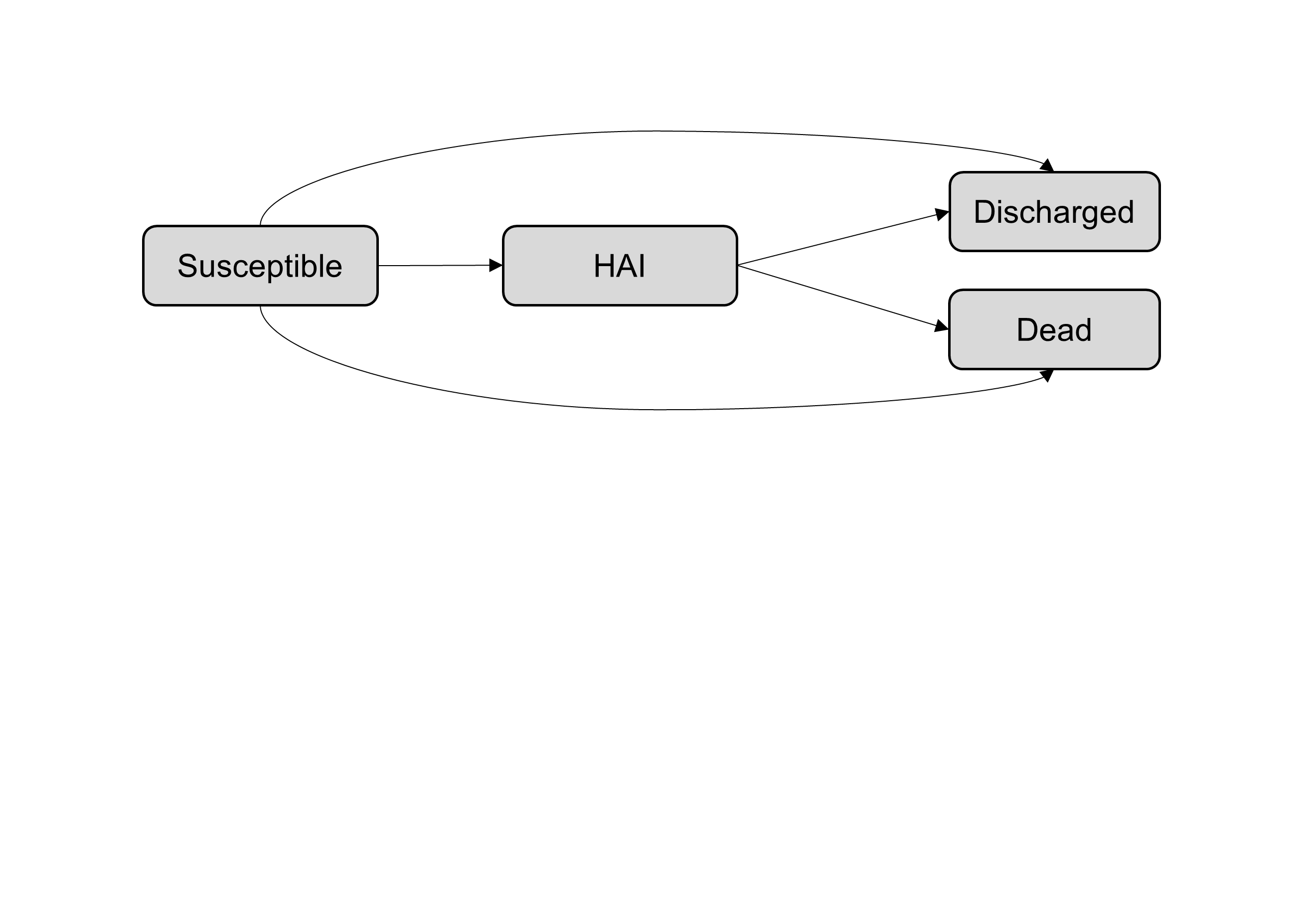

Supplement: Morris et al. supplementary material 2 — Morris et al. supplementary material [file S0899823X24000953sup002.tif]

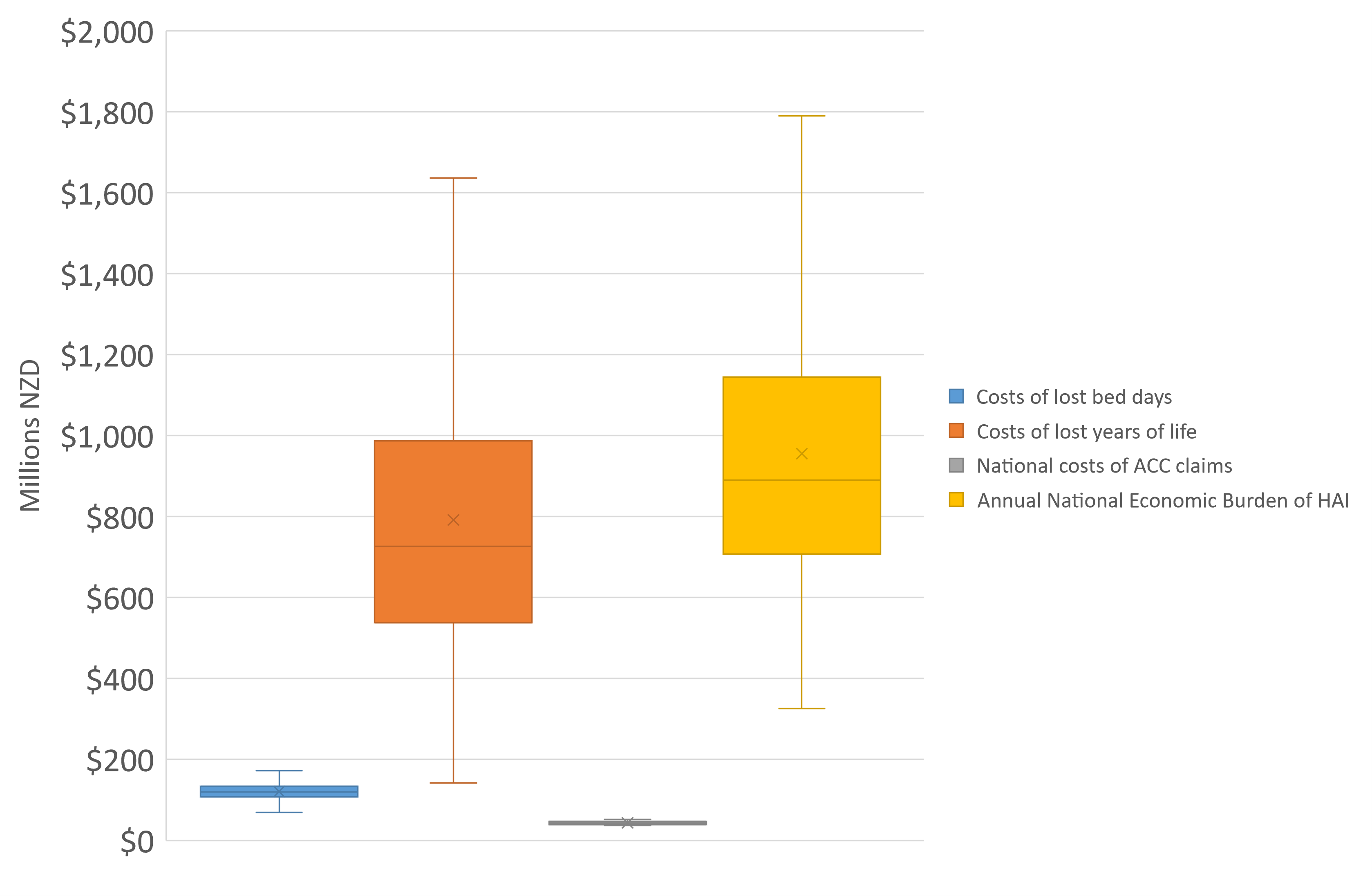

Supplement: Morris et al. supplementary material 3 — Morris et al. supplementary material [file S0899823X24000953sup003.tif]
